# Clinicians’ Perceived Understanding of Biostatistical Results in the Medical Literature: A Cross-Sectional Study

**DOI:** 10.3390/medicina55060227

**Published:** 2019-05-30

**Authors:** Kurubaran Ganasegeran, Alan Swee Hock Ch’ng, Mohd Fadzly Amar Jamil, Irene Looi

**Affiliations:** 1 Clinical Research Center, Seberang Jaya Hospital, Ministry of Health Malaysia, Jalan Tun Hussein Onn, 13700 Seberang Jaya, Penang, Malaysia; alanchng@yahoo.com (A.S.H.C.); fadzly.crc@gmail.com (M.F.A.J.); irenelooi@yahoo.com (I.L.); 2 Department of Medicine, Seberang Jaya Hospital, Jalan Tun Hussein Onn, 13700 Seberang Jaya, Penang, Malaysia

**Keywords:** biostatistics, medical literature, appraisal, clinicians, evidence-based medicine

## Abstract

*Background and objectives:* The continuum of evidence-based medicine (EBM) depends solely on clinicians’ commitment to keep current with the latest clinical information. Exploration on clinicians’ understanding of biostatistical results in the medical literature is sparse to date. This study aimed to evaluate clinicians’ perceived understanding of biostatistical results in the medical literature and the factors influencing them. *Materials and Methods:* A cross-sectional study was conducted among 201 clinicians at the Seberang Jaya Hospital, a cluster-lead research hospital in Northern Malaysia. A self-administered questionnaire that consisted of items on sociodemographics, validated items on clinicians’ confidence level in interpreting statistical concepts, perceived understanding of biostatistics, and familiarity with different statistical methods were used. Descriptive, univariate, and multivariate analyses were conducted. *Results:* Perceived understanding of biostatistical results among clinicians in our sample was nearly 75%. In the final regression model, perceived understanding was significantly higher among clinicians who were able to interpret *p*-values with complete confidence (AOR = 3.0, 95% CI 1.1–8.1), clinicians who regularly encounter measures of central tendencies (AOR = 2.3, 95% CI 1.1–5.2), and clinicians who regularly encounter inferential statistics (AOR = 2.2, 95% CI 1.1–4.5) while appraising the medical literature. *Conclusions:* High perceived understanding was significantly associated with clinicians’ confidence in interpreting statistical concepts and familiarity with different statistical methods. Our findings form a platform to understand clinicians’ ability to appraise rigorous biostatistical results in the medical literature for the retrieval of evidence-based data to be used in routine clinical practice.

## 1. Introduction

In keeping abreast with the latest clinical information and practice guidelines, modern medical practice has driven clinicians to constantly seek the best available evidence-based summaries that emerged from original research reports [1]. Retrieving “evidence data” relies heavily on the critical appraisal of the literature that originates from the fields of epidemiology and biostatistics [2]. The evolving rigor of biostatistics from elementary to advanced statistical methodology reporting in medical literature has cautioned clinicians to carefully apprehend and adopt core data of research to be applied in patient care [1].

While medical publishing has shown a commensurate increase in open access models, caveats of misleading statistics or erroneous results data have been identified within the scholarly literature which, if adopted in routine clinical practice, could affect unintended consequences in patient care [3,4,5]. It has become a scientific responsibility for readers who regularly appraise medical literature to identify and reject such errors. Such a phenomenon has generated greater pressure to clinicians with little or no formal understanding in biostatistics and epidemiology to not only struggle in interpreting research results accurately but also identify the statistical power, statistical significance or wrong statistical concepts being employed in medical literature [1,6].

Preliminary studies that were conducted at the beginning of the evidence-based medicine (EBM) era between 1980s and 1990s found poor understanding of basic statistical concepts amongst clinicians when appraising medical literature [7,8,9,10]. With biostatistical models showing greater complexities, recent investigations have enumerated much a lower understanding and limited abilities amongst clinicians to interpret the results of research findings confidently [6,11]. A cross-sectional study among medical residents from the USA found that 41.4% of the residents were able to understand the results reported in the medical literature, and their ability to apprehend statistical concepts was significantly associated with male gender, having advanced degrees or having prior statistical training [6]. That study also highlighted that most residents were able to interpret relative risk but were less likely to know correct interpretation of odds ratios from regression models [6]. A study amongst Greek medical residents highlighted that residents’ perceived familiarity ratings of biostatistical concepts like standard deviations, *p*-values, confidence intervals and correlation coefficients were predictive of better knowledge interpretability of biostatistical results [1]. A survey from Thailand that explored clinicians’ knowledge level of interpreting biostatistical results found that specialists and clinicians who had previously attended statistical courses were more likely to have better knowledge of interpreting results in the medical literature [10]. A recent survey among obstetrics and gynecology residents showed little or no confidence in interpreting research statistics and that confidence level was better with increased seniority, as well as among those having previous publications or who had attended prior epidemiology or statistical courses [12]. A Malaysian study concluded that most clinicians lack basic biostatistical knowledge, and almost half of them do not appraise journal articles regularly due to possible lack of confidence in interpreting different statistical concepts reported in the literature [13].

The continuum of medical knowledge is based on contemporary epidemiological and biostatistical methods. Primary objectives of clinical studies often summarize baseline characteristics of the intervention and control groups using descriptive statistics such as central tendencies or measures of dispersion and succinctly compare the efficacy of novel drugs or interventions that catalyzes statistical significance through inferential statistics like t-test, Pearson’s chi-square or Fisher’s exact tests [14,15]. Du Prel and colleagues, through their systematic review of 1828 publications from six medical journals across general medicine, obstetrics and gynecology, and emergency medicine, concluded that familiarities in common descriptive statistics and inferential statistical tests were sufficient for a clinician to interpret results in at least 70% of articles in the medical literature [14].

The bulk of literature has highlighted poor understanding and confidence level amongst clinicians to effectively interpret biostatistical results to make applied decisions in patient care [9,15,16,17,18]. With low statistical understanding, clinicians’ recommendations and interventions can be influenced by framing (e.g., mortality vs. survival rates) or nontransparent risk measures interpretation (e.g., relative risks), alarming clinicians on potential difficulties to provide the best evidence-based care to their patients [19]. Most studies conducted to date used medical residents or postgraduate trainees rather than practicing clinicians who apply EBM for patient care. While a single Malaysian study explored clinicians’ biostatistics use, this study was rather descriptive in nature, with no possible inferences deduced to find exact causality or associations that may have influenced clinicians’ understanding of biostatistical results in medical literature. To highlight the current lacunae of knowledge and confidence gap, this preliminary analytical investigation from Malaysia aims to evaluate clinicians’ perceived understanding of biostatistical results in the medical literature and the factors influencing them, ranging from demographics, statistical confidence, and frequency of different statistical data encounters.

## 2. Materials and Methods

### 2.1. Study Setting and Design

This single-center cross-sectional study was conducted between January–June 2018 among 234 clinicians at the Seberang Jaya Hospital, a cluster-lead research hospital located in mainland Penang, Northern Malaysia. The hospital’s Clinical Research Centre (CRC) was tasked with conducting various clinical trials and epidemiological studies in Northern Malaysia, apart from organizing annual EBM and Introduction to Clinical Research (ICR) training workshops, with module exposures on biostatistics [20]. All clinicians (medical officers and clinical specialists) from the medical, surgical, and its allied departments were invited to participate in the study during Departmental Continuous Medical Education (CME) sessions. Permissions and assistance from the departmental heads were obtained.

### 2.2. Ethical Issues

This study was conducted in accordance with the Helsinki Declaration. The research protocol was approved by the Medical Research Ethics Committee (MREC), Ministry of Health Malaysia (government approval number: NMRR-18-64-39559 IIR). The objectives and benefits of the study were explained verbally and in written form attached to the questionnaires. Participants were assured that information obtained would be confidential and their participation would be anonymous. A written consent was obtained from those who agreed to participate.

### 2.3. Study Instrument

All clinicians completed a self-administered questionnaire in English, given our sample cohort preferred communicating in English in line with their routine clinical practice. The questionnaire consisted of four parts. In the first part, five questions on sociodemographics that included items on gender, age, clinician level (defined as clinical specialists or medical officers according to profession grades as legislated by the Malaysian Public Service Department [21]), highest biostatistics education, and previous research experience were included. The second part included four validated items that assessed confidence on the interpretation of statistical concepts adapted from Windish and colleagues [6]. These questions were scored on a five-point Likert scale in which “1” indicated “none” and “5” indicated “complete confidence”. These items were dichotomized into two categories, “complete confidence” and “less confidence” to ease interpretation. Perceived understanding was defined as the perception and ability to correctly apprehend the statistical terms and results of medical literature [6]. The perceived understanding was assessed with a single dichotomous question, “Do you understand the results and statistical terms when appraising medical literature?” with response options “Yes” or “No”. The final part evaluated types of statistical data frequently encountered in medical literature. The evaluation utilized the adaptation and the modification of the scale developed by Rashid and Subramaniam [13] regarding the frequency of use of different statistical data. Six subdomains with 27 items included major statistical data, namely: (1) Data organization (tables, bar graph, pie chart, histogram, frequency polygon, dot plot, box and whisker plot, stem and leaf plot, scatter plot); (2) measures of central tendency (mean, median, mode); (3) measures of dispersion (range, percentiles, quartiles, interquartile, variance, standard deviation); (4) inferential statistics (hypothesis testing, probability, t-test, ANOVA, chi-square); (5) correlation and dispersion (correlation, linear regression, logistic regression); and (6) measuring scales. These items were scored on a five-point Likert scale ranging from “1” never to “5” always. Total subdomain scores were tabulated and dichotomized into two categories, “irregularly” and “regularly” according to mean cut-off points to ease interpretation (mean cut-offs utilized as total scores for each subdomain were normally distributed).

### 2.4. Pilot Testing and Internal Consistency

The questionnaire was pilot-tested by 15 clinicians who were nonparticipants of the study. There were no issues with the administration of the questionnaire in terms of clarity, relevance or comprehension of the items in the instrument. Three participants expressed difficulties with the wordings of certain items in the instrument, and this issue was resolved by revising the words accordingly. The questionnaire was then finalized. The time required to complete the questionnaire was approximately 15 min. Cronbach’s alpha co-efficient of the four items scale that evaluated confidence level on the interpretation of statistical concepts was 0.91, indicating an excellent internal consistency.

### 2.5. Data Analysis

**Figure 1:**
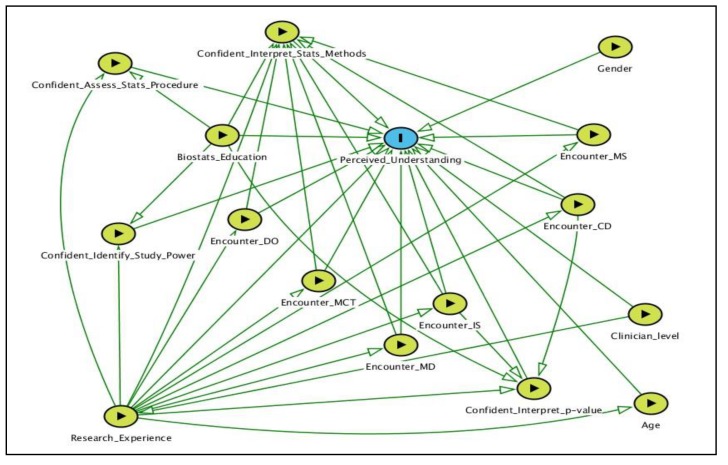
A DAG of the factors of interest in the analysis. Outcome variable is identified as blue oval with black frame; exposures are identified as green oval with black frame. DO: Data organization; MCT: Measures of central tendency; MD: Measures of dispersion; IS: Inferential statistics; CD: Correlation and dispersion; MS: Measuring scales; DAG: Directed acyclic graph.

A framework was constructed to visualize the associations between variables of interest using the directed acyclic graph (DAG) method, plotted by DAGitty (http://dagitty.net/) [22] (Figure 1). Data collected were analyzed using SPSS version 23.0. All quantitative data were found to be normally distributed using statistical and graphical methods. Descriptive statistics were conducted for all variables. Chi-square test was used to assess the associations between perceived understanding and categorical variables. Multiple logistic regression analysis using Enter, Forward, and Backward regression techniques were employed to determine the factors associated with perceived understanding of biostatistical results in the medical literature among clinicians. Variable selection into the regression models was based on statistical significance (*p* < 0.05) at univariate level as well as principles of parsimony, model fitness, and biological plausibility. Assumptions of logistic regression, including multicollinearity between independent variables, were tested and met. The most parsimonious final multivariate model was selected and presented. Statistical significance was set at *p* < 0.05.

## 3. Results

### 3.1. Sample Characteristics

Table 1 shows sample characteristics. Two hundred and thirty-four clinicians were invited to participate in the study, and 201 (85.9% response rate) participated. Thirty-three clinicians declined participation due to the following reasons: Unwilling to volunteer participation due to lack of interest, 13 (5.5%), time constraints, 10 (4.3%), and being on-call/attending emergency at the time of study, 10 (4.3%). Our sample consisted of 77 (38.3%) men and 124 (61.7%) women. The mean (±SD) age of the clinicians was 32 (±5) years, and the age ranged between 26 and 54 years old. Most clinicians were older than thirty years (111 (55.2%)), with 163 (81.1%) medical officers and 167 (83.1%) having attained highest level of biostatistics education in medical school. More than half of the clinicians in our sample had previous research experience (106 (52.7%)), and the majority of them were perceived to understand biostatistical results in the medical literature (149 (74.1%)). When appraising results of medical literature, most clinicians had complete confidence in their ability to interpret *p*-values for a given result (159 (79.1%)), interpret results of a statistical method used (184 (91.5%)), and identify factors that influence the study power (175 (87.1%)). However, only twelve (6%) of the clinicians had complete confidence in their ability to assess if correct statistical procedures were used to answer research questions (Table 1).

### 3.2. Types of Data Frequently Encountered in Medical Literature

Table 2 exhibits types of data frequently encountered in medical literature. Inferential statistics was the most regularly encountered (128 (63.7%)), followed by data organization (118 (58.7%)), correlation and dispersion (108 (53.7%)), measures of central tendency (92 (45.8%)), measures of dispersion (87 (43.3%)), and measuring scales (68 (33.8%)).

### 3.3. Association Between Sample Characteristics and Perceived Understanding of Biostatistical Results in the Medical Literature

Table 3 shows the association between sample characteristics and clinicians’ perceived understanding of biostatistical results in the medical literature. Perceived understanding was significantly higher among clinicians aged more than 30 years old (OR = 8.8, 95% CI 4.1–19.1), clinical specialists (OR = 2.7, 95% CI 1.1–7.3), clinicians interpreting *p*-values for a given result with complete confidence (OR = 4.1, 95% CI 1.6–10.4), clinicians interpreting results of statistical methods used with complete confidence (OR = 2.8, 95% CI 1.1–7.8), and clinicians identifying factors that influence the study power with complete confidence (OR = 2.4, 95% CI 1.1–5.6).

### 3.4. Association between Types of Data Encountered and Perceived Understanding of Biostatistical Results in the Medical Literature

Table 4 exhibits the association between types of data encountered and perceived understanding of biostatistical results in the medical literature. Perceived understanding was significantly higher among clinicians who regularly encountered measures of central tendency (OR = 2.1, 95% CI 1.1–4.0), inferential statistics (OR = 2.2, 95% CI 1.1–4.1), and measuring scales (OR = 2.3, 95% CI 1.1–4.9).

### 3.5. Factors Associated With Perceived Understanding of Biostatistical Results in the Medical Literature by Multiple Logistic Regression Analysis

In the multiple logistic regression analysis (Backward Wald regression), interpreting *p*-values for a given result, regular encounter with measures of central tendency, and regular encounter with inferential statistics were significantly associated with clinicians’ perceived understanding of biostatistical results in the medical literature. Clinicians interpreting *p*-values for a given result with complete confidence had significantly higher perceived understanding as compared to clinicians with lesser confidence (AOR = 3.0, 95% CI 1.1–8.1). Clinicians who regularly encountered measures of central tendency had a significantly higher perceived understanding as compared to clinicians with irregular encounter with such measures (AOR = 2.3, 95% CI 1.1–5.2). Clinicians who regularly encountered inferential statistics had a significantly higher perceived understanding as compared to clinicians with irregular encounter with such measures (AOR = 2.2, 95% CI 1.1–4.5). The total model was significant (*p* < 0.001) and accounted for 24% of the variance. There was no multicollinearity between independent variables (Table 5).

## 4. Discussion

This study aimed to determine the factors associated with clinicians’ perceived understanding of biostatistical results in the medical literature. Of the 201 clinicians surveyed, almost 75% were perceived to understand biostatistical results reported in the medical literature. Perceived understanding of biostatistical results in this study was slightly lower than that found among medical students in Spain who underwent training in epidemiology and biostatistics (80–90%) [23], but relatively higher than that found among emergency medicine residents in the USA (38%) [17], government hospital doctors in Malaysia (29.2%) [13], postgraduate medical students in India (38.1%) [24], and resident physicians in Saudi Arabia (33%) [25]. The relatively higher frequency of perceived understanding among clinicians in this study could be attributed to the exposures received through training workshops (EBM and ICR) and consultations conducted continuously at our center that critically emphasizes different statistical techniques and epidemiological methods to effectively interpret the literature. In the final regression model, clinicians’ complete confidence in interpreting *p*-values and regular encounters with measures of central tendency and inferential statistics were significantly associated with perceived understanding of biostatistical results in the medical literature.

While medical research aims to testify to the effects of treatment efficacy or describe relationships between hypothesized attributes by quantifying measures of mean difference, risk ratios, odds ratios or correlation parameters, readers often objectively rely on “statistical significance” of the observed effect through yielded *p*-values of <0.05 that rejects the null hypothesis (Ho) through inferential statistical tests (e.g., t-test or χ^2^ tests) [26]. Our findings showed that most clinicians (91.5%) had complete confidence in interpreting *p*-values for a given result, which is still the most significant predictor to influence perceived understanding of biostatistical results. This finding was consistent with previous work conducted among anesthetists in the UK [27], OMS residents in the USA [28], and medical residents in Greece [1]. While global trends of interpreting medical literature have raised unprecedented concerns on the reliability of *p*-values that offsets information on the magnitude or importance of the effect, researchers were called to be unified to provide plausible estimates about the magnitude of the effect on the population from which the data were sampled, yet to yield the observed effect to be “clinically important” [12]. As these arguments pose substantial justifications to evidence-based practitioners, mixed findings emerged in the current sample. In this study, clinicians’ regular encounters with measures of central tendency and inferential statistics were significantly associated with perceived understanding of biostatistical results, consistent with previous investigations [1,12]. The measure of central tendency is the single most crucial value that speculates on and comprehends the distribution of clinical and demographic data in a particular sample. These measures provide a platform of apprehending descriptive data to subsequently make inferences through hypothesis testing [29]. Intriguingly, measures of dispersion (variability) such as standard deviations, which are important to describe the spread of the data around the center values, showed no statistical significance with perceived understanding in the final regression model. Most standardized effect size calculations that aim to provide an alternative to *p*-values involve a version of the standard deviation of the outcome measure as a denominator [30]. As such measures showed no statistical significance, it could be postulated that the current sample of clinicians in this study were similarly persistent in line with global trends of championing “statistical significance” of *p*-values rather than “clinical importance” of effect sizes.

The association between gender and perceived understanding of biostatistical results showed mixed variations in previous investigations. While the present finding was consistent with most literature that found no statistical difference between gender and perceived understanding of biostatistical results [24,31], few studies found contrary outcomes, with two from the USA and Saudi Arabia concluding that men have significantly higher odds of biostatistical results understanding compared to women [6,25], while one study from Pakistan identified women to be more likely to understand the results of medical literature [32]. Seniority has been reported to influence biostatistical results understanding among healthcare workers. This study found that older clinicians were more likely to understand results of the medical literature. The finding was consistent with the work conducted in India [24] and Ottawa [12] but different to that reported in Greece [1] and Saudi Arabia [25].

Nonspecialist clinicians were postulated to have greater understanding of biostatistical results due to greater pressure to manage a wider spectrum of clinical cases, prompting them to regularly appraise medical literature to retrieve latest medical knowledge for applied patient care [25]. However, our sample showed that clinical specialists were more likely to have greater understanding of biostatistical results as compared to medical officers, corroborating with previous investigations in the USA [6] and India [24], but inconsistent with a recent evidence from Saudi Arabia [25]. A plausible explanation for the occurrence of this current phenomenon could be pressure against clinical specialists to conduct research and explore novel scientific evidence for applied patient care within their subject matter expertise to accelerate the continuum of evidence-based practice as part of their key performance index.

Although clinicians obtained basic biostatistics knowledge during professional training, they are not apt to confidently use their knowledge to interpret the results of different statistical methods used in the medical literature for applied decision making [33]. The ability to identify the test used for each particular situation is influenced by the data measurement scale, number of groups, relationship between participants (independent groups or related), and the researcher’s intention to establish associations or causal relationships between groups [33]. As noted in the regression model, although the interpretation of results of a statistical method used was included in the model, it was relatively marginal and nonsignificant (*p* = 0.059). A similar finding was noted in a previous study [28]. Given its near significance *p*-value, the predictor might have achieved statistical significance in association with perceived understanding, and the plausibility for such a phenomenon to occur could be attributed to the size of our sample, which may have increased the possibility of type II error in our analysis.

Certain limitations of this study should be acknowledged. The cross-sectional nature of the current study from a single hospital in Northern Malaysia could not establish causal relationships between independent variables and limits the generalizability of the study findings. Despite these limitations, the novelty found from this preliminary hypothesis generating survey could set the stage for future testable hypotheses using robust methodological techniques to explore possible causal relationships of critical variables, such as gender, age, specialties, confidence level of interpreting results of a statistical method, identifying study power, and familiarity of different statistical techniques used that may influence clinicians’ perceived understanding of biostatistical results. We embarked on cautious reporting by using the term “perceived understanding” rather than “actual understanding”, as our study was not powered to do so. The foundation of using the term “perceived understanding” lies within the nature of the primary outcome measure (dependent variable) used in this study. Although the outcome measure was adapted from previous literature, it was a single item with a binary measure (yes/no response) and not a scale which underwent a psychometric evaluation to capture the “actual understanding” of biostatistical results among clinicians. However, “understanding” could itself reflect a change over time, not a state at a particular moment in time, and it could be influenced by the processes of continuous practice, learning, and skills acquirement. At this preliminary level of investigation, it is important for us to know if clinicians at least think or believe that they understand biostatistics (a reflection and introspection) through self-reported measures capturing the process of understanding, rather than to conclude their “actual understanding” identified by rigorous measurement tools which are yet to be available. There is a trend which could be observed in the literature that “perceived understanding” was relatively high among clinicians who attended a biostatistical course or training [6,23]. The current study showed similar consistencies, given that our center organizes annual EBM and ICR training workshops with module exposures and consultations in biostatistics for clinicians. However, the hyper-inflated rate reported in this study as compared to previous literature could be attributed to the term used, “perceived understanding” rather than “actual understanding”, which could have a predilection of respondents’ overconfidence of self-reported measures. To offset these limitations, we recommend that future studies inspect respondents’ veracity through response subjectivity (open-ended question responses) to synthesize the exact situation of understanding biostatistical results in the medical literature. However, this approach may face challenges while dealing with a cohort of clinicians who may decline participation due to time constraints, lack of interest, and the stressful nature of clinical practice. Further, we anticipate this statistically-driven concept research paper to face debates on the concerns of dichotomizing certain continuous variables into categorical ones. While categorization could cause potential loss of variation to a variable, or to a larger extent causing harmful effects for a biological type of variables’ interpretability, categorization of certain variables in this study could have minimal effect, with emphasis on justifications to be based solely on the principles for real life interpretations. For example, the categorization of age ≤30 or >30 years in this study accurately reflects the current situation in Malaysian clinical practice to postulate our study outcomes, whereby clinicians aged ≤30 years are mostly novice clinicians who may have just embarked into their actual service years post-residentship, while those clinicians aged >30 years are more senior ones according to their profession grades, in which involvement in research and retrieving evidence-based data according to their specialty or subject matter expertise would be part of their key performance indexes.

## 5. Conclusions

The relatively high perceived understanding of biostatistical results in this study was associated with clinicians’ complete confidence in interpreting *p*-values and regular encounters with measures of central tendencies and inferential statistics. Although these findings enhance our knowledge on the factors influencing “perceived understanding” of results reported in the medical literature, exploration of “correct understanding” through respondents’ subjectivity should be further evaluated, as apprehension of the rigorousness of contemporary biostatistical concepts currently used in the medical literature is needed for the continuum of accurate “evidence-based appraisal” to be applied to patient care.

## Figures and Tables

**Table 1:** Sample characteristics (*n* = 201).

Characteristics	*n* (%)
Gender	
Men	77 (38.3)
Women	124 (61.7)
Age group (years)	
≤30	90 (44.8)
>30	111 (55.2)
Clinician level	
Medical officer	163 (81.1)
Clinical specialists	38 (18.9)
Highest biostatistics education	
Medical school	167 (83.1)
Postgraduate degree	34 (16.9)
Previous research experience	
No	95 (47.3)
Yes	106 (52.7)
Interpret *p*-values for a given result	
Less confidence	42 (20.9)
Complete confidence	159 (79.1)
Interpret results of a statistical method used	
Less confidence	17 (8.5)
Complete confidence	184 (91.5)
Assess if correct statistical procedure was used to answer research questions	
Less confidence	189 (94.0)
Complete confidence	12 (6.0)
Identify factors that influence study power	
Less confidence	26 (12.9)
Complete confidence	175 (87.1)
Perceived understanding of biostatistical results in the medical literature	
No	52 (25.9)
Yes	149 (74.1)

**Table 2:** Types of data frequently encountered (*n* = 201).

Characteristics	*n* (%)
Data organization	
Irregularly	83 (41.3)
Regularly	118 (58.7)
Measures of central tendency	
Irregularly	109 (54.2)
Regularly	92 (45.8)
Measures of dispersion	
Irregularly	114 (56.7)
Regularly	87 (43.3)
Inferential statistics	
Irregularly	73 (36.3)
Regularly	128 (63.7)
Correlation and dispersion	
Irregularly	93 (46.3)
Regularly	108 (53.7)
Measuring scales	
Irregularly	133 (66.2)
Regularly	68 (33.8)

**Table 3:** Association between sample characteristics and perceived understanding of biostatistical results in the medical literature (*n* = 201).

Characteristics	Perceived Understanding of Biostatistical Results in the Medical Literature		OR (95% CI)
	Yes *n* (%)	No *n* (%)	
Gender			
Men	56 (72.7)	21 (27.3)	1 1.1 (0.6–2.1)
Women	93 (75.0)	31 (25.0)	
Age group (years)			
≤30	48 (53.3)	42 (46.7)	1 8.8 (4.1–19.1)*
>30	101 (90.1)	10 (8.9)	
Clinician level			
Medical officer	116 (71.2)	47 (28.8)	1 2.7 (1.1–7.3)*
Clinical specialists	33 (86.8)	5 (13.2)	
Highest biostatistics education			
Medical school	122 (73.1)	45 (26.9)	1 1.4 (0.6–3.5)
Postgraduate degree	27 (79.4)	7 (20.6)	
Previous research experience			
No	67 (70.5)	28 (29.5)	1 1.4 (0.8–2.7)
Yes	82 (77.4)	24 (22.6)	
Interpret *p*-values for a given result			
Less confidence	20 (47.6)	22 (52.4)	1
Complete confidence	129 (81.1)	30 (18.9)	4.1 (1.6–10.4)*
Interpret results of a statistical method used			
Less confidence	9 (52.9)	8 (47.1)	1 2.8 (1.1–7.8)*
Complete confidence	140 (76.1)	44 (23.9)	
Assess if correct statistical procedure was used to answer research questions			
Less confidence	140 (74.1)	49 (25.9)	1 1.1 (0.3–4.0)
Complete confidence	9 (75.0)	3 (25.0)	
Identify factors that influence study power			
Less confidence	15 (57.7)	11 (42.3)	1 2.4 (1.1–5.6)*
Complete confidence	134 (76.6)	41 (23.4)	

* Statistically significant (*p* < 0.05).

**Table 4:** Association between types of data encountered and perceived understanding of biostatistical results in the medical literature.

Types of Data	Perceived Understanding of Biostatistical Results in the Medical Literature		OR (95% CI)
	Yes *n* (%)	No *n* (%)	
Data organization			
Irregularly	57 (68.7)	26 (31.3)	1 1.6 (0.9–3.1)
Regularly	92 (78.0)	26 (22.0)	
Measures of central tendency			
Irregularly	74 (67.9)	35 (32.1)	1 2.1 (1.1–4.0)*
Regularly	75 (81.5)	17 (18.5)	
Measures of dispersion			
Irregularly	79 (69.3)	35 (30.7)	1 1.8 (0.9–3.5)
Regularly	70 (80.5)	17 (19.5)	
Inferential statistics			
Irregularly	47 (64.4)	26 (35.6)	1 2.2 (1.1–4.1)*
Regularly	102 (79.7)	26 (20.3)	
Correlation & dispersion			
Irregularly	66 (71.0)	27 (29.0)	1 1.4 (0.7–2.6)
Regularly	83 (76.9)	25 (23.1)	
Measuring scales			
Irregularly	92 (69.2)	41 (30.8)	1 2.3 (1.1–4.9)*
Regularly	57 (83.8)	11 (16.2)	

* Statistically significant (*p* < 0.05).

**Table 5:** Multiple logistic regression analysis (Backward Wald); factors associated with perceived understanding of biostatistical results in the medical literature.

Characteristics	B	SE	Wald	AOR (95% CI)
Interpret *p*-values for a given result				
Less confidence	Ref	Ref	Ref	Ref 3.0 (1.1–8.1)*
Complete confidence	−1.1	0.5	5.0	
Interpret results of a statistical method used				
Less confidence	Ref	Ref	Ref	Ref 3.1 (0.9–9.8)
Complete confidence	−1.1	0.6	3.6	
Measures of central tendency				
Irregularly	Ref	Ref	Ref	Ref 2.3 (1.1–5.2)*
Regularly	0.9	0.4	4.4	
Inferential statistics				
Irregularly	Ref	Ref	Ref	Ref 2.2 (1.1–4.5)*
Regularly	0.8	0.4	4.3	

* Statistically significant (*p* < 0.05). Note: Variables entered include all significant variables in the univariate analysis. Variable “interpret results of a statistical method used” was a marginal predictor (*p* = 0.059). AOR: Adjusted odds ratio; CI: Confidence interval.
